# Accuracy and Precision Assessment of AoA-Based Indoor Positioning Systems Using Infrastructure Lighting and a Position-Sensitive Detector

**DOI:** 10.3390/s20185359

**Published:** 2020-09-18

**Authors:** Álvaro De-La-Llana-Calvo, David Salido-Monzú, José-Luis Lázaro-Galilea, Alfredo Gardel-Vicente, Ignacio Bravo-Muñoz, Borja Rubiano-Muriel

**Affiliations:** 1 Department of Electronics, University of Alcalá, Alcalá de Henares, 28801 Madrid, Spain; josel.lazaro@uah.es (J.-L.L.-G.); alfredo.gardel@uah.es (A.G.-V.); ignacio.bravo@uah.es (I.B.-M.); borja.rubiano@edu.uah.es (B.R.-M.); 2 Institute of Geodesy and Photogrammetry, ETH Zürich, 8093 Zürich, Switzerland; david.salido@geod.baug.ethz.ch

**Keywords:** Indoor Positioning System (IPS), Visible Light Positioning (VLP), Position Sensitive Detector (PSD), Angle of Arrival (AoA), accuracy, precision

## Abstract

Unlike GNSS-based outdoor positioning, there is no technological alternative for Indoor Positioning Systems (IPSs) that generally stands out from the others. In indoor contexts, the measurement technologies and localization strategies to be used depend strongly on the application requirements and are complementary to each other. In this work, we present an optical IPS based on a Position-Sensitive Detector (PSD) and exploiting illumination infrastructure to determine the target position by Angle of Arrival (AoA) measurements. We combine the proposed IPS with different positioning strategies depending on the number of visible emitters (one, two, or more) and available prior or additional information about the scenario and target. The accuracy and precision of the proposal is assessed experimentally for the different strategies in a 2.47 m high space covering approximately 2.2 m^2^, using high-end geodetic equipment to establish the reference ground truth. When the orientation of the target is known from external measurements, an average positioning error of 8.2 mm is obtained using the signal received from only one emitter. Using simultaneous observations from two emitters, an average positioning error of 9.4 mm is obtained without external information when the target movement is restricted to a plane. Conversely, if four signals are available, an average positioning error of 4.9 cm is demonstrated, yielding the complete 3D pose of the target free of any prior assumption or additional measurements. In all cases, a precision (2σ) better than 5.9 mm is achieved across the complete test space for an integration time of 10 ms. The proposed system represents a prospectively useful alternative for indoor positioning applications requiring fast and reliable cm-level accuracy with moderate cost when smart illumination infrastructure is available in the environment.

## 1. Introduction

It is a well-established and proven fact that GNSS-based outdoor positioning systems for tracking and navigation are reliable and easily integrated into real-time applications. These systems, however, cannot be used in indoors applications with the same performance and results due to the non-uniform degradation of the satellite signals and the multipath effect. The results of the unsatisfactory use of GNSS signals indoors led to the emergence of many proposals in the last two decades.

There are plenty of research studies that propose Indoor Positioning Systems (IPSs) based on multiple sensors, mostly inertial ones, to simultaneous locate and map (SLAM) a device using referenced beacons. Thus, we can find different proposals of IPSs to help people in daily tasks [1], to smoothly position users while moving from outdoor to indoor environments [2], in indoor localization in a hospital environment [3], in intelligent logistics applications [4], in indoor transport tasks [5], etc. As shown in several papers [6,7,8,9], indoor positioning systems have been around for a long time, and there are sectors in which they are widely used in everyday activities, providing applications with significant added value.

For the development of indoor localization systems, different techniques have been used, many of which are complementary and can operate in a cooperative manner, aiming at better positioning results. The choice of which particular system or technology to use depends on the application and positioning scenario. When determining what type of system to develop and what technologies to use, factors such as energy consumption, coverage, accuracy, privacy, and cost will be key [10,11,12].

Different research works that make use of LED-based lighting also provide positioning services. Papers such as [13,14] propose the use of indoor Visible Light Communication (VLC) technologies to send positioning information to devices connected within the covered area. LEDs used in VLC systems function as beacons and lighting. The state-of-the-art of IPSs exploiting visible light was comprehensively reviewed in [13], covering the most relevant works in this field classified according to technology and measurement principle. Focusing on the works most closely related to the present proposal—namely AoA—and image-based positioning, Reference [13] showed that the achieved accuracy and precision for such techniques are typically in the order of 5 to 10 cm. In [15], each lighting LED sends spatial coordinates in 3D, and an image sensor receives the signal with an accuracy of around 1.5 m. In [16], the authors used an image sensor with an image sampling frequency of up to 48 kHz. The receiver decodes the information about the referenced position of the LED lighting, and knowing the impact point in the image sensor, the positioning is obtained. In the tests carried out within an area of 5.4 × 7.5 m with a ceiling height of 3 m, the maximum error in the horizontal plane was 10 cm. Other authors such as Huynh and Yoo [17] performed the positioning using more LEDs (four) at the same time, achieving positioning errors below 10 cm. In [18], the development of an IPS based on Received Signal Strength (RSS) from the LEDs was enhanced by using multiple photodiodes in the receivers. The results obtained showed an average positioning error of 20 cm over an effective positioning range of 1.8 m. Zachar et al. [19] proposed systems with Infrared emitters (IRED) and IR cameras, optimized for the detection of IRED beacons. Following the example of increasing the number of LEDs in transmission, other authors have proposed using several photodiodes in the receiving device. For example, in [18], an LED transmission grid and multiple photodiode receivers determined the position with an average error of 20 cm over an area of 1.8 square meters.To increase the coverage area, Zachar et al. [19] changed the transmission and receiving devices from the visible to the infrared range. Reference [20] proposed a triangulation-based system, where moving agents send signals in the non-visible range so that several photodiodes arranged in the environment obtain the angle of arrival to the emitters. For a large test area of 7 × 2 square meters, errors of up to 70 cm were obtained. In [21], a work based on two infrared light emitters and three photodetectors positioning a robot in an indoor environment was presented. The sensors do not incorporate lenses, and the achieved positioning provides an accuracy of 10 cm and 0.1 rad for a robot moving at 0.2 m/s. It is worth noting that besides the PSD, detection can be combined with Kalman filtering including certain prior knowledge on possible trajectories to follow a mobile agent. Reference [22], for example, assumed that the agent moves along a horizontal plane achieving maximum and average localization errors of 8.97 cm and 1.97 cm, respectively.

The paper is organized as follows: First an overview of previous research underlying this work is provided in Section 2. In Section 3, the proposed IPS, based on optical signals and a PSD sensor, is described, discussing the different positioning strategies depending on the number of visible emitters. Section 4 describes the setup of the experimental evaluation. The results of the accuracy and precision assessment of the proposal are provided in Section 5. Finally, conclusions and the outlook are summarized in Section 6.

## 2. Background

The research group to which the authors of the article belong has extensive experience in investigating IPS systems based on infrared and visible light. Multiple localization systems have been proposed based on measurements of the phase shift and angle of arrival of the signals reaching the PSD.

In [23], an IPS based on the Angle of Arrival (AoA) was proposed assuming that the mobile to be positioned moves along a plane. The mobile incorporates the emitting beacon and the PSD, located in a known position in the environment, and retrieves the positioning of the mobile from the AoA measurements. The advantages of this PSD-IPS system when compared to similar configurations based on the phase shift or using non-positioning-sensitive detectors are higher accuracy and precision due to the continuous resolution along the PSD surface and larger coverage area. The main shortcomings, on the other hand, are the need for dedicated infrastructure, i.e., installing the PSD on the ceiling, and errors due to multipath effects and distortions that affect the ideal AoA measurement. A complete analysis and correction of these distortions was presented in [24,25]. A procedure to model and correct the errors due to multipath effects was proposed in [26,27,28]. From those previous works, it can be concluded that PSD-based IPSs using AoA observations provide better positioning performance than implementations using phase differences. Work [29] presented an analysis of the more convenient multiple access discrimination techniques for the development of PSD-based VLP. It was therein shown that Frequency-Division Multiple-Access (FDMA) is the most suitable technique for such systems. FDMA allows discriminating between the different emitters of the system with almost no interference, unlike Code-Division Multiple-Access (CDMA), which introduces significant errors when obtaining the point of impact due to code cross-correlation interferences. Reference [30] presented solutions for the implementation of the PSD-based IPS described in [29] using only a system-on-chip microcontroller.

Related to previous works based on PSD sensors used to develop positioning systems, it is worth noting [22], which assumed that the PSD is fixed on the ceiling of the environment and the emitter is onboard the agent that moves in a horizontal plane.

This work combines the use of the proposed IPS with different positioning strategies (using the signal received from only one LED emitter, the signal received from two LED emitters, and the signal received from four or more LED emitters) to empirically determine the accuracy and precision achievable by this IPS based on optical signals and AoA. The main contributions of this work are:

- The system configuration places the PSD onboard the mobile agent and the emitters on fixed positions in the environment. This configuration allows for exploiting lighting infrastructure for positioning while enabling full 3D pose estimation otherwise unfeasible using a single emitter. Inverting the configuration, however, increases the complexity of the positioning since the PSD orientation can no longer be pre-calibrated, but has to be continuously estimated.
- Localization strategies for different numbers of received signals are proposed so that the system can be adapted to varying coverage requirements and application needs.
- The accuracy and precision of the proposed system are experimentally assessed for various configurations regarding the number of received signals.
- A much higher measurement rate can be obtained compared to that of image-based sensors.
- The achieved accuracy is higher than most previous existing IPS alternatives, whose typical performance is of tens of centimeters up to one meter.
- The proposed system does not require any information from previous points to obtain good accuracy.
- Our proposal is more stable in relation to certain factors such as power variation and ambient light than optical RSS-based systems and, unlike such solutions, can operate using a single detector.

## 3. IPS Proposal Based on the Optical Signal with a PSD Sensor

**Figure 1:**
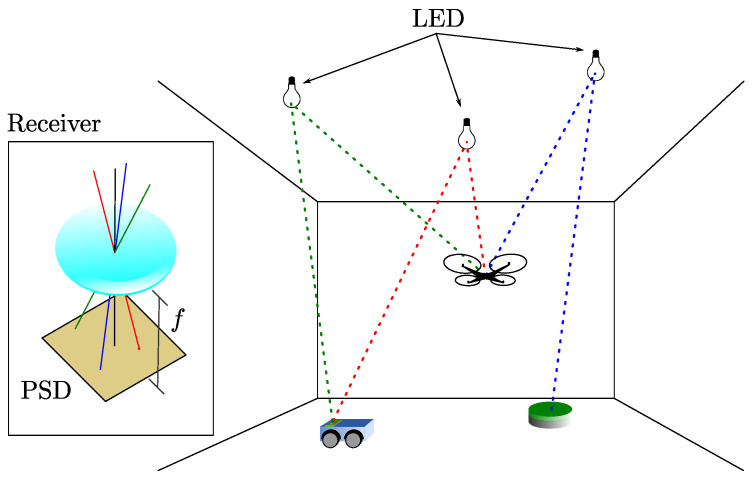
Diagram of the proposed positioning system based on optical AoA measurements with a PSD sensor.

The scheme of the proposed positioning system is shown in Figure 1. The LED emitters are placed on the ceiling in fixed known positions, each one emitting an intensity modulated sinusoidal signal of different frequency that allows their identification via an FDMA scheme. The signals are received simultaneously by the PSD sensor, and accounting for the optical system mounted on the PSD, the AoA for each emitter is calculated. As discussed in Section 2, this AoA-based strategy provides higher immunity to multipath effects. As can be seen, the positioning scheme is the opposite of [23]. In this case, we add the problem of having to know in each position the orientation of the receiver, unlike in [23], where this information was fixed and known. It should be noted that the emitters can be considered punctual and have a Lambertian emission pattern with rotational symmetry, making their orientation irrelevant for AoA measurements; note that this would not be the case for an RSS-based localization strategy.

In this work, we use a PSD sensor as the receiver. The PSD sensor is a type of photodiode that consists of four anodes and a common cathode.

The signal reception point on the PSD surface based on the four electric currents generated at each PSD anode can be calculated according to Equations (1) and (2).

(1) $$\begin{array}{c} x=\frac{L_{X}}{2}\frac{\left(V_{X2}+V_{Y1}\right)-\left(V_{X1}+V_{Y2}\right)}{V_{X1}+V_{X2}+V_{Y1}+V_{Y2}}, \end{array}$$

(2) $$\begin{array}{c} y=\frac{L_{Y}}{2}\frac{\left(V_{X2}+V_{Y2}\right)-\left(V_{X1}+V_{Y1}\right)}{V_{X1}+V_{X2}+V_{Y1}+V_{Y2}}, \end{array}$$

where $V_{X1},V_{X2},V_{Y1},$ and $V_{Y2}$ are the voltages of each of the four anodes of the PSD sensor. The PSD output currents must be previously amplified using transimpedance amplifiers because these signals are very low, and considerable error could be generated when digitizing them. It should be noted that $V_{X1},V_{X2},V_{Y1},$ and $V_{Y2}$ are the voltages that have been corrected by an electrical calibration [24] to compensate gain errors due to drift and tolerance of the electronic components of the amplifiers. The constants $L_{X}$ and $L_{Y}$ are the PSD sensor dimensions.

**Figure 2:**
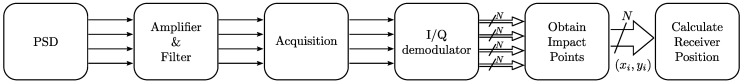
Flowchart of the positioning pipeline.

Figure 2 shows a flowchart summarizing the processing pipeline to compute the receiver position from the signals detected by the PSD. The four output photocurrents of the PSD are amplified and filtered to limit the signal bandwidth to that of the emitters. The conditioned signals are then digitized and the corresponding sequences processed by In-phase and Quadrature (I/Q) demodulation to obtain the module of the signal corresponding to each of the *N* emitters. The outputs of this sub-stage are therefore 4N signal module values (a detailed description of this stage is provided in [29]). Using Equations (1) and (2), the *N* impact points $\left(x_{i},y_{i}\right)$—one per emitter—are then computed from the 4N module values. The received position is finally calculated from the impact points $\left(x_{i},y_{i}\right)$, as described next.

The LEDs are assumed coplanar and their position perfectly known; the impact of deviations from these assumptions are discussed later. The specific localization procedure varies depending on the number of emitters within the Field of View (FoV) of the receiver. Our focus herein is not to discuss possible applications, but to determine the performance implications of each strategy.

- One emitter within the FoV: In this case, to obtain the position, the orientation of the PSD should be known, and the PSD should move on a plane parallel to the one of the emitters. If the motion plane is not parallel, the position could still be calculated given a previous estimation of the rotation and translation relation between both planes. This case would be applicable, e.g., to a mobile robot navigating on a fixed plane (floor) and whose orientation is obtained by an external system. This external system could be based on an Inertial Measurement Unit (IMU) providing gyroscopic measurements for angular dead-reckoning. The main problem in such a case would be the cumulative error of inertial systems, making it only feasible for short distances between calibration points where the position and orientation are reset with another external system.
- Two emitters within the FoV: This situation requires the motion plane of the PSD to be parallel to the emitters’ plane. In this case, the distance between both planes can be calculated from the measurements. With two emitters, the rotation of the PSD over its surface vector can be obtained, but the other angles defining the total orientation of the PSD must be known. This case would cover most applications where it is necessary to position a mobile robot, since it allows obtaining by itself the position and rotation of the robot.
- Three or more emitters within the FoV: In this case, the 3D coordinates and orientation of the PSD can be obtained without any previous knowledge of the motion space. This case would cover all applications where it is necessary to know all the angles and position of the agent without limiting possible solutions, such as Unmanned Aerial Vehicle (UAV) localization.

The model of the system formed by the PSD and the lens is based on a pinhole model. The relationship between the 3D coordinates of the emitter $\left(X_{e},Y_{e},Z_{e}\right)$ and the 2D coordinates of the impact point on the PSD surface $\left(x_{i}^{{}^{\prime }},y_{i}^{{}^{\prime }}\right)$ is given by:

(3) $$\left(\begin{array}{c} sx_{i}^{{}^{\prime }}\\ sy_{i}^{{}^{\prime }}\\ s \end{array}\right)=\underset{A}{\underbrace{\left(\begin{array}{ccc} f & 0 & C_{x}\\ 0 & f & C_{y}\\ 0 & 0 & 1 \end{array}\right)}}\underset{R}{\underbrace{\left(\begin{array}{ccc} r_{11} & r_{12} & r_{13}\\ r_{21} & r_{22} & r_{23}\\ r_{31} & r_{32} & r_{33} \end{array}\right)}}\left(\begin{array}{c} X_{e}-X_{r}\\ Y_{e}-Y_{r}\\ Z_{e}-Z_{r} \end{array}\right),$$

where *s* is the scaling factor of the 3D-to-2D projection, *f* is the lens focal length, and $C_{x}$ and $C_{y}$ are the coordinates of its optical center.

The intrinsic matrix A is defined by the lens parameters *f*, $C_{x}$, and $C_{y}$, which can be obtained from a geometrical calibration of the system [25]. The computation of the 2D coordinates of the impact point on the PSD surface can only be carried out once the electrical corrections and the radial and tangential distortion corrections of the system formed by the PSD and the lens have been made [24,25]. The extrinsic matrix R matrix is calculated from the three Euler angles that define the orientation of the PSD sensor with respect to the world coordinate system.

### 3.1. Positioning with Only One Emitter

When the PSD sensor only receives the signal from one emitter, the position of the PSD can be obtained as long as the PSD moves in a plane and the orientations of the PSD are known, so that the angles that define R are known. We assume for simplicity that the plane of PSD sensor surface and the motion plane are coplanar and define the reference system so that this plane can be expressed as Z=constant.

Knowing the values of the matrices A and R, the position $\left(x_{r},y_{r},z_{r}\right)$ of the PSD sensor can be obtained from the measured impact point $\left(x_{i},y_{i}\right)$ on its surface as:

(4) $$\left(\begin{array}{c} X_{r}\\ Y_{r}\\ Z_{r} \end{array}\right)=-R^{-1}A^{-1}\left(\begin{array}{c} x_{i}\\ y_{i}\\ 1 \end{array}\right)s+\left(\begin{array}{c} X_{e}\\ Y_{e}\\ Z_{e} \end{array}\right).$$

Defining the column vector q as:

(5) $$q=\left(\begin{array}{c} q_{1}\\ q_{2}\\ q_{3} \end{array}\right)=-R^{-1}A^{-1}\left(\begin{array}{c} x_{i}\\ y_{i}\\ 1 \end{array}\right)$$

we obtain:

(6) $$\left(\begin{array}{c} X_{r}\\ Y_{r}\\ Z_{r} \end{array}\right)=qs+\left(\begin{array}{c} X_{e}\\ Y_{e}\\ Z_{e} \end{array}\right),$$

where the value of $Z_{r}$ is known from the movement plane of the PSD sensor. The scaling factor *s* can then be obtained as:

(7) $$s=\frac{Z_{r}-Z_{e}}{q_{3}},$$

where $q_{3}$ is the third element of q. Knowing *s*, the receiver coordinates can be calculated from Equation (6).

### 3.2. Positioning with Two Emitters

The derivation for this case also relies on the PSD surface and motion plane, defined as Z = constant, being coplanar. When the PSD sensor receives the signal from two emitters, its rotation relative to the surface vector can be calculated in addition to its position. If the emitters’ plane is parallel to the motion plane, the separation between the emitters’ plane and the motion plane of the receiver can be obtained from the measures.

**Figure 3:**
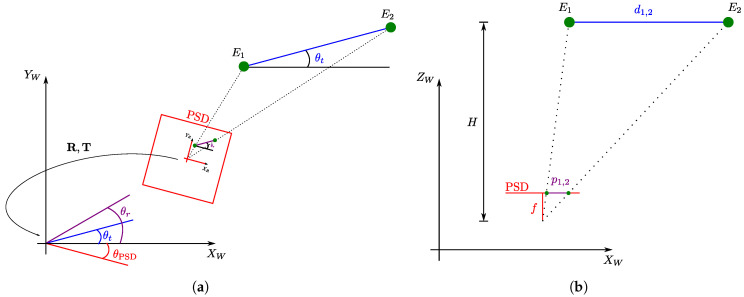
(**a**) Method for obtaining the angle *θ*_PSD_. (**b**) Method for obtaining the H separation between the emitter plane and the PSD plane.

The rotation of the PSD is computed from the coordinates $\left(X_{e_{1}},Y_{e_{1}},Z_{e_{1}}\right)$ and $\left(X_{e_{2}},Y_{e_{2}},Z_{e_{2}}\right)$ of the two emitters in the world reference system and the coordinates $\left(x_{i_{1}},y_{i_{1}}\right)$ and $\left(x_{i_{2}},y_{i_{2}}\right)$ of the point of impact on the PSD surface for each emitter. The variables involved in these computations are depicted in Figure 3a.

The angle between the two emitters with respect to the *x* axis of the world reference system is:

(8) $$\theta_{t}=\mathrm{atan}2\left(\frac{Y_{e_{1}}-Y_{e_{2}}}{X_{e_{1}}-X_{e_{2}}}\right),$$

where atan2 is the arctangent function producing a modulus 2π output by using the sign of the numerator and denominator to solve quadrant ambiguities. Similarly, the angle between the impact points of the two emitters on the surface of the PSD with respect to the *x* axis of the PSD reference system is computed as:

(9) $$\theta_{r}=\mathrm{atan}2\left(\frac{y_{i_{1}}-y_{i_{2}}}{x_{i_{1}}-x_{i_{2}}}\right).$$

Once these angles are obtained, the rotation between the world reference system and the PSD can be calculated as:

(10) $$\theta_{\mathrm{PSD}}=\theta_{t}-\theta_{r},$$

from which the rotation matrix R can be obtained. From R, the position of the PSD can be obtained similarly to the previous case, using the coordinates of Emitter 1.

(11) $$\left(\begin{array}{c} X_{r}\\ Y_{r}\\ Z_{r} \end{array}\right)=-R^{-1}A^{-1}\left(\begin{array}{c} x_{i_{1}}\\ y_{i_{1}}\\ 1 \end{array}\right)s+\left(\begin{array}{c} X_{e_{1}}\\ Y_{e_{1}}\\ Z_{e_{1}} \end{array}\right)$$

In this case, the plane where the receiver is located is unknown, so Equation (7) cannot be used directly to obtain the scaling factor. However, since the focal length *f* and the separation between the emitters in the world and between their corresponding impact point on the surface of the PSD are known, the height *H* that separates the planes of the emitters and the PSD can be obtained as depicted in Figure 3b according to:

(12) $$H=f\frac{d_{1,2}}{p_{1,2}}=f\frac{\sqrt{{\left(X_{e_{1}}-X_{e_{2}}\right)}^{2}+{\left(Y_{e_{1}}-Y_{e_{2}}\right)}^{2}}}{\sqrt{{\left(x_{i_{1}}-x_{i_{2}}\right)}^{2}+{\left(y_{i_{1}}-y_{i_{2}}\right)}^{2}}}.$$

The scaling factor can then be calculated knowing the separation *H* between the planes as:

(13) $$s=\frac{Z_{e}-H-Z_{e}}{q_{3}}=\frac{-H}{q_{3}}$$

Once the scale factor is obtained, the position of the PSD can be calculated from (11).

### 3.3. Positioning with Three or More Emitters

In situations when the receiver moves in a coplanar plane to the emitters’ plane, using more than two emitters would enable reducing random error contributions, exploiting the overdetermined system with some optimization approach. In situations when the full PSD pose, i.e., 3D coordinates and three orientation angles, is required, an established computer vision strategy can be applied. Such a strategy is known as the Perspective-n-Point (PnP) problem and allows determining the orientation and position of a calibrated camera given a set of *n* 3D points in the world and their corresponding 2D projections in the image.

There are several methods to obtain the pose, from linear methods such as Direct Linear Transformation (DLT) [31] to more complex methods, each with certain restrictions, such as PosIt [32], Coplanar PosIt [33], or CamPoseCalib(CPC) [34]. In this work, we use a joint approach based on DLT and CPC. DLT is a linear method that allows obtaining the pose with the information of four emitters, and CPC is an iterative method based on the Gauss–Newton algorithm and non-linear least-squares optimization.

While DLT cannot directly provide an accurate solution due to the positioning problem being non-linear, it can be used to obtain a roughly approximated solution free of any initialization. Such a solution can in turn be used to initialize the CPC algorithm, increasing its robustness by avoiding possible local minima in the iterative process.

## 4. Experimental Setup

The objective of the experimental tests is to evaluate the proposed IPS under different conditions by varying the receiver positions for different emitter configurations. To assess the accuracy and precision of the proposal, the results are compared with a more accurate system based on high-end geodetic total stations.

**Figure 4:**
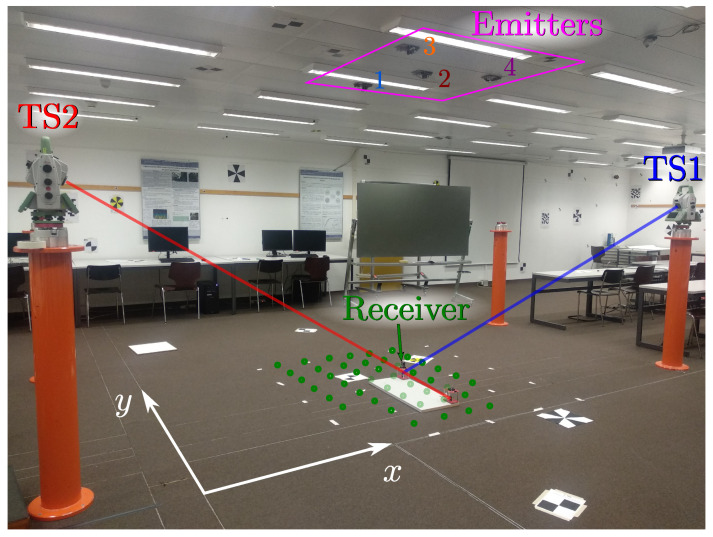
Test environment.

Figure 4 shows a picture of the test environment. Four emitters were located on the ceiling (magenta square), and the PSD sensor was placed at several positions on the floor (green points). The ground truth was derived from the joint measurements from two total stations tracking points connected rigidly with the PSD.

**Figure 5:**
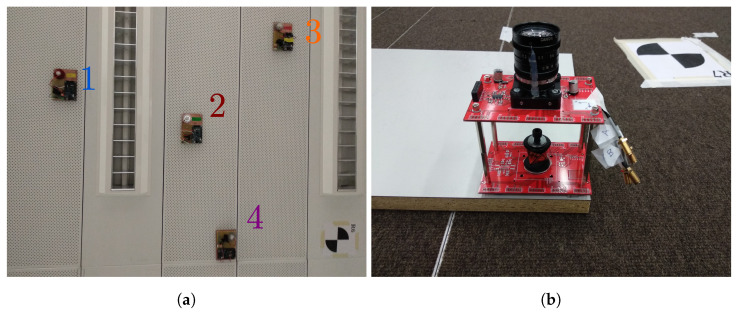
(**a**) Emitters used for the experimental evaluation as placed. View of their placement on the lab ceiling. (**b**) Receiver (PSD and lens system) and reference mini prism.

Figure 5a shows the emitters placed on the ceiling. The emitter used for the tests is an LED with 3 W of average emitted power and a Lambertian emission diagram. The selection of suitable emitters for the proposed system requires taking into account the emitted power and distribution, as well as wavelength coupling with the detector sensitivity, so that the expected SNR in certain scenarios and positions can be estimated taking into account the properties of the PSD (active area and spectral efficiency) and the lens system. The positions of the emitters were selected as a trade-off between coverage and geometrical strength for the triangulation computations; larger separation would increase the latter, reducing error propagation from the AoA measurements to the estimated positions, but in turn, reducing the area where all emitters are simultaneously within the FoV of the receiver. The emitters were modulated with 6, 8, 10, and 14 kHz. We chose these frequencies so that none shared a multiplicity relationship in order to reduce the interference due to possible harmonics. The four emitters were used to analyze the results in the different configurations discussed in Section 3. For receiver positions, the signal of the 4 emitters was acquired, and depending on the analyzed configuration, a subset formed by 1, 2, or 4 of them was used.

The receiver, shown in Figure 5b, is composed of a Hamamatsu PSD sensor S5991-01 (information and datasheet at: https://www.hamamatsu.com/eu/en/product/type/S5991-01/index.html) (nominal active area: 9×9 mm^2^, measured bandwidth: 200 kHz) and the corresponding analog processing stages for transimpedance conversion and amplification. The filtering stage was designed to limit the signal bandwidth to that of the 4 emitters’ modulations. This, together with the emitter modulation frequencies selected to be sufficiently separated from those typically present in artificial lighting, enables minimizing the impact of ambient artificial illumination on the measurements. A Canon lens JF7.5 1.4 was mounted on the PSD to form the image of the ceiling of the PSD-sensitive area. The lens was selected to balance the trade-off between distortion, FoV, and multipath interferences; a shorter focal lengthwould increase coverage, but also introduce higher accuracy errors due to the coupling of more multipath components from nearby walls and objects while increasing distortion. In this case, the most relevant aspect in the lens selection was the system coverage, so lenses with the appropriate focal lengthfor the desired coverage should be shortlisted and the larger one among the commercially available candidates selected to maximize collected power. Once selected, the expected energy projected onto the PSD considering the lens effective area can be computed to adjust the gain of the conditioning stage accordingly to maximize the SNR. If the distortion introduced by the selected lens is unacceptably large, its compensation can be addressed as proposed in [25]. The signal of each of the anodes of the PSD after being amplified was acquired with the NI9239 acquisition board with a sampling rate of 50 kSamples/s and a resolution of 20 bits. To measure the position and orientation of the receiver with the reference total stations, this was mounted on a rigid base together with two 360 mini prisms (Leica GRZ101). One of the prisms was placed directly in the vertical of the PSD center (see Figure 5b), while the other was separated horizontally from this by approximately 65 cm. Both prisms maintained their relative position on the base, which allowed calculating the absolute rotation of the PSD.

The receiver was placed at 44 positions on the floor plane in an area of approximately 1.25 × 1.75 m^2^. This area was centered on Emitter 2 and dimensioned so that the signal from such emitter across all possible test positions covered the entire surface of the PSD.

Aiming at collecting enough data for evaluating both precision and accuracy properly, time series of 15 s were acquired statically on each position. This data length enables sufficient reduction of random contributions via averaging for the accuracy assessment while also providing enough data for a statistical analysis of random errors.

Two Leica TS60 (information and datasheet at: https://leica-geosystems.com/en-us/products/total-stations/robotic-total-stations/leica-nova-ts60) total stations were used to obtain the ground truth of the emitter and receiver positions. The Leica TS60 provides a nominal angular accuracy of 0.5” and a nominal distance accuracy of 0.6 mm + 1 ppm. The total stations were placed on fixed pillars within the test environment and their absolute positions calculated by resection from measurements to three prisms places on other pillars whose position was known from previous measurement campaigns. The overdetermined resection process also provides metrics on the precision of the total stations positioning. The estimated 1σ deviations are 0.2 mm in the *x* and *y* axes and 0.5 mm in the *z* axis.

**Figure 6:**
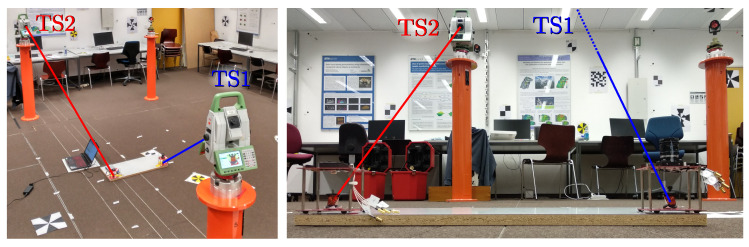
Receiver targeted by the total stations.

As can be seen in Figure 6, each of the total stations measures the position of a different prism. TS1measures the prism colocated with the receiver, providing directly the position of the receiver after a vertical correction with a fixed value measured mechanically. TS2 measures the position of the other prism, which is used together with the first one to calculate the rotation of the receiver. The positions of the emitters were also derived from the total stations with an estimated accuracy of 5 mm using reflectorless measurements.

**Figure 7:**
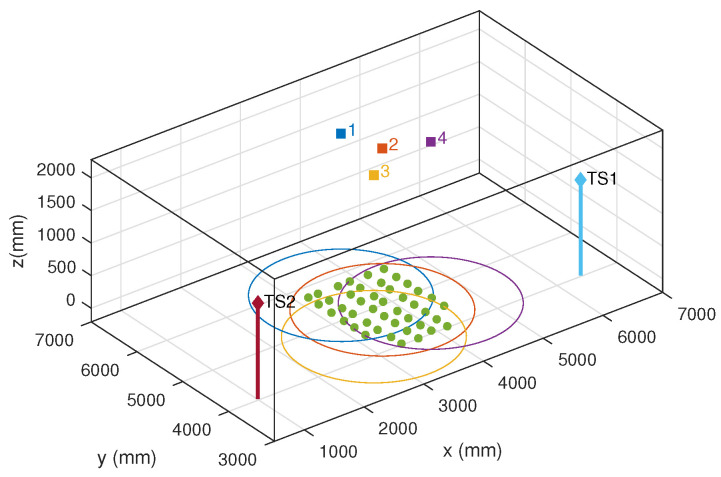
Positions of the emitters, receivers (green), and total stations in the test environment.

Figure 7 shows the positions of the receiver (green points) and the emitters within the environment along with the positions of the total stations used to obtain the ground truth. The circles of different colors represent the area where the PSD receives the signal from the corresponding emitter. The radius *R* of the circle is calculated as R=(l∗H)/f, where *H* is the vertical separation between emitter and receivers, *f* the lens focal length, and *l* the radius of the effective sensitive area of the PSD. It was measured experimentally by evaluating the area of the PSD where the detected signal reliably represented the optical signal incident angle. The measured value (3.5 mm) was smaller than the nominal sensitive area of the PSD (4.5 mm), indicating the impact of border effects when the incident footprint does not lie completely within the sensitive area.

The measured coordinates of the emitters along with their corresponding modulation frequency are indicated in Table 1.

It should be noted that there are very small differences in the *z* coordinate of the receiver positions. When positioning with one emitter, the positioning plane is calculated considering the average height of all positions of the receivers. This introduces an error smaller than 2 mm in the final position. The maximum vertical deviation between emitter positions is 4 mm, so when positioning with two emitters, those are assumed coplanar, which introduces an error in the height determination of approximately 0.1 mm, whose impact on the final position is negligible.

In the next section, the positioning results are compared with the ground truth obtained with the total station.

The positioning error is calculated as follows. The coordinates of the receiver position $p_{i}$ obtained with the total stations are defined as:

(14) $$p_{i}=\left(X_{r_{i}},Y_{r_{i}},Z_{r_{i}}\right)$$

where *i* is the index of the receiver positions. The receiver position ${\hat{p}}_{i}$ as estimated by the IPS is defined as:

(15) $${\hat{p}}_{i}=\left({\hat{X}}_{r_{i}},{\hat{Y}}_{r_{i}},{\hat{Z}}_{r_{i}}\right)$$

The positioning error $e_{i}$ is then calculated as the euclidean distance between those coordinates as:

(16) $$e_{i}=\sqrt{{\left(X_{r_{i}}-{\hat{X}}_{r_{i}}\right)}^{2}+{\left(Y_{r_{i}}-{\hat{Y}}_{r_{i}}\right)}^{2}+{\left(Z_{r_{i}}-{\hat{Z}}_{r_{i}}\right)}^{2}}$$

The mean positioning error $\bar{e}$ and standard deviation $\sigma_{e}$ are calculated considering all measured positions as:

(17) $$\bar{e}=\frac{1}{N}\sum_{i=1}^{N}e_{i}$$

where *N* is the number of receiver positions, and:

(18) $$\sigma_{e}=\sqrt{\frac{1}{N}\sum_{i=1}^{N}{\left(e_{i}-\bar{e}\right)}^{2}}$$

The mean, standard deviation, and maximum values of the positioning error were used to evaluate the performance of the proposed IPS depending on the number of the emitters in the FoV of the receiver.

## 5. Results

In this section, the empirical results obtained with the proposed IPS using the experimental setup described above are shown. The performance assessment of the proposal includes an evaluation of the accuracy for different configurations depending on the number of emitters within the FoV of the receiver (Section 5.1) and an evaluation of the precision as a function of the measurement time (Section 5.2).

### 5.1. Accuracy Assessment

Positioning accuracy was evaluated by using large integration times making random errors negligible in relation to systematic ones. The positioning results shown below were obtained by integrating over 15 s of signal, which was experimentally proven to provide sufficient absorption of random variations.

#### 5.1.1. Positioning with Only One Emitter

**Figure 8:**
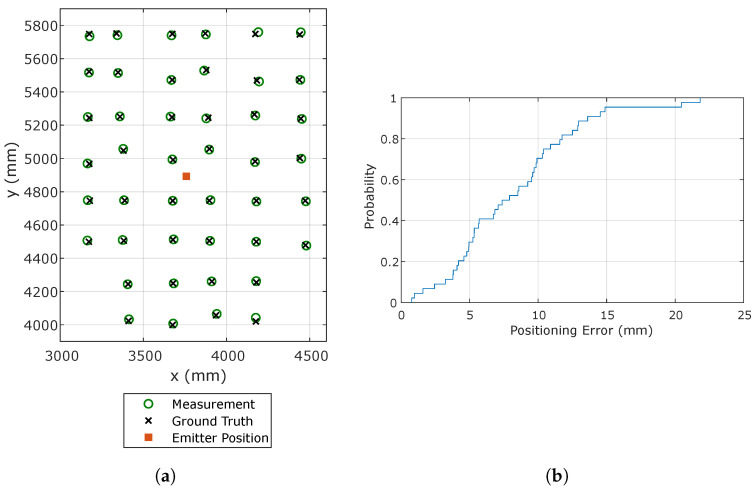
Positioning results using Emitter 2. (**a**) Estimated and true receiver positions; (**b**) CDF of the positioning error.

When positioning using only one receiver, the rotation of the PSD and the distance between the emitter and the PSD movement plane should be known. For this assessment, the rotation and distance were measured with the total stations. Emitter 2 was used because it was centered on the positions where the receiver was moved. Figure 8a shows the positions obtained together with the ground truth.

Table 2 shows the mean, standard deviation, and maximum values of the errors for Emitter 2 and 3, to assess the performance both when the emitter is in the center (No. 2) and in a corner (No. 3) of the evaluated area. The performance using the other corner emitters yields very similar figures to those from No. 3 due to the symmetry of the setup.Note that, since random contributions are largely absorbed by averaging, the standard deviation represents mostly the spatial variability of the positioning error. When using Emitter 2, the positioning errors are smaller than 2 cm for all the evaluated positions. When using an emitter located in a corner, the errors are degraded between 30% and 65%. This is caused by more positions of the test grid illuminating the sensor closer to the edge of its sensitive area, where it is more subject to distortions and non-linearities, hence less accurate.The achieved accuracy is better than 12 mm in 80% of the cases, as can be seen from the Cumulative Distribution Function (CDF) of the positioning error in Figure 8b, and the mean error is 8.20 mm. It should be noted, however, that these results rely on perfect prior information on the receiver rotation and the distance between the emitter and the PSD movement plane. In a real application, these parameters need to be measured externally. While the latter can be easily measured with high accuracy on typical environments, the estimation of the receiver rotation cannot be easily obtained with high accuracy, and errors in measuring such rotation will directly couple to positioning errors. In order to quantify this impact, emulations adding AWGN to the rotation measurements were carried out, resulting in an increase of the mean positioning error of 5%, 22%, 55%, and 170% when adding noise levels with standard deviations of 0.1°, 0.5°, 1°, and 2°, respectively. The overall expected accuracy should therefore be evaluated also taking into account the expected performance of the rotation measurement system.

#### 5.1.2. Positioning with Two Emitters

The evaluation of positioning accuracy using two emitters was carried out using a fixed pair of emitters. Better results could be achieved in a real application by optimizing the selected pair inline based on some meaningful criteria such as geometrical strength to reduce error propagation or distance to the receiver to maximize SNR.

**Figure 9:**
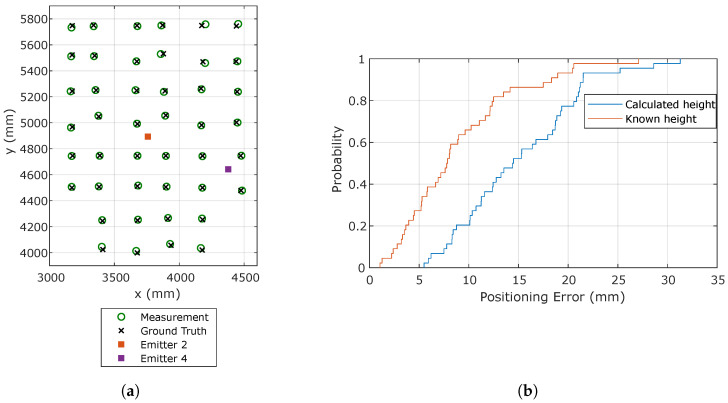
Positioning results using Emitters 2 and 4. (**a**) Estimated and true receiver positions using calculated height; (**b**) CDF of the positioning error.

When the signals from two emitters are simultaneously available, it is possible to calculate the receiver rotation and the height *H* between the plane of the emitters and the receiver moving plane by assuming that both are co-planar, which is reasonable in many typical positioning scenarios. Providing an accurate external measurement of the height *H* can, however, improve the positioning accuracy. Since such a height can usually be measured easily, the positioning error was computed for both cases, i.e., when the emitter height is calculated from the measurements or known in advance. Figure 9a,b shows the estimated and true positions along with the CDF of the positioning error.

Table 3 shows the mean, maximum, and standard deviation of the positioning errors for both examined cases and for the two emitter configurations: one emitter in the center and one in a corner of the test area and the two emitters in corners. The results of the other possible emitter combinations are similar due to the symmetry of the setup. The accuracy improvement when the height is known, due to the fixed z coordinate in the estimation, is clearly visible. When the height is not known, an accuracy better than 21 mm in 80% of the cases is obtained. Note that, although worse than the previous results using only one emitter, this positioning strategy does not require any additional measurement.

**Figure 10:**
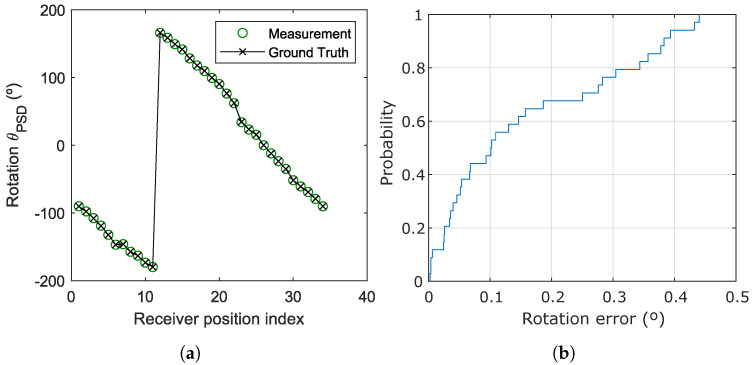
Rotation angle results using Emitters 1 and 4. (**a**) Calculation of the rotation angle; (**b**) CDF of the rotation error.

An additional test was carried out to evaluate specifically the accuracy of the rotation measurement. The receiver was placed under Emitter 2 and rotated 34 angles. Emitters 1 and 4, having a more favorable geometry, were used to compute the rotation. Figure 10a shows the measured rotation values $\theta_{\mathrm{PSD}}$ (Equation (10)) together with the ground truth data, while Figure 10b shows the CDF of the rotation error.

The mean, standard deviation, and maximum error in the determination of the rotation angles are detailed in Table 4. As can be seen, the error in the measurement of the rotation is below 0.35° in 80% of the cases, with an average value of 0.157°.

#### 5.1.3. Positioning with Three or More Emitters

The positioning results for more than three emitters were calculated using all the available constellation in the experimental setup with four emitters. This configuration allows obtaining the total pose of the receiver, i.e., the 3D coordinates of its position and the three Euler angles that define its orientation. Note that, if the specific application does not require full 3D pose information, the data from three or more emitters can also be used in an over-determined approach on both of the previously described positioning strategies if their associated restrictions (e.g., planar target movement) can be applied, the redundancy thus enabling better accuracy.

**Figure 11:**
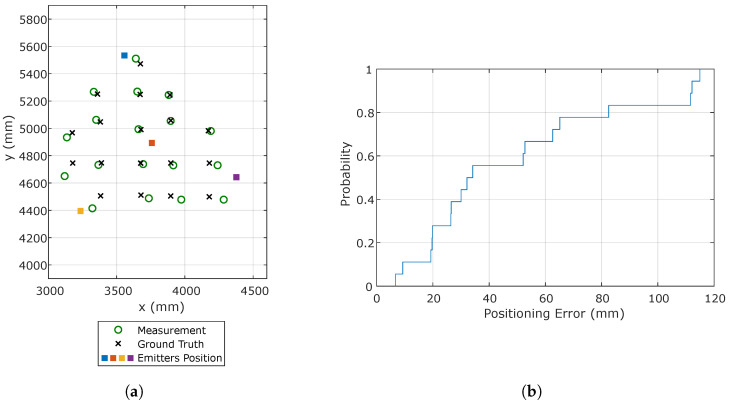
Positioning results using the information from the four emitters with CamPoseCalib(CPC). (**a**) Calculation of the receiver positions; (**b**) CDF of the positioning error.

Since the receiver does not see all the emitters in all the test positions, only those where all the emitters are within the receiver FoV were analyzed. Figure 11a shows the results by adjusting with the CPC [34] algorithm along with the ground truth, and Figure 11b depicts the CDF of the positioning error.

It can be seen how in this case that the errors are greater than in the previous cases using one and two emitters. This is expected due to the increase in the number of estimates (three coordinates and three angles) using information from only four emitters. Small errors in the determination of the impact point of any of the emitters introduces high errors in the determination of the pose. If signals from more than four emitters were available, the increased redundancy would enable better results.

As in the previous cases, the mean, standard deviation, and maximum values of the positioning errors are shown in Table 5. The average error is smaller than 4.9 cm, and in 80% of the cases, the errors are smaller than 8.3 cm. Note that these results rely only on prior information on the emitter positions, with no additional knowledge or assumptions on the receiver movement.

### 5.2. Precision Assessment

The results were first analyzed depending on the measurement time used to obtain the point of impact. For each of the positions of the receiver, the different impact points were obtained for various integration times. The covariance matrix of these impact points was then calculated in each case, providing information on the error ellipsoid of each position.

In Appendix A, some parameters obtained from the covariance matrix, such as the trace, determinant, and maximum eigenvalues, are analyzed as a function of the integration time.

To provide some meaningful quantity to interpret the precision results, the semi-major axis of the ellipsoid containing 95% of the points for each position was analyzed. The semi-major axis is calculated as $a=\sqrt{5.991\lambda_{1}}$ where $\lambda_{1}$ is the highest eigenvalue.

**Figure 12:**
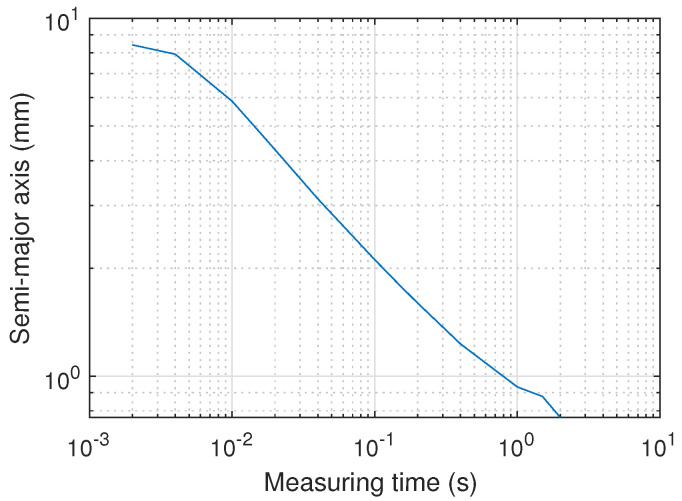
Mean value of the semi-major axis of the error ellipsoid projected on the plane of movement as a function of measurement time.

Figure 12 shows the mean value of all the semi-major axes of the ellipsoids projected to the plane of motion as a function of the measurement time. The projection is made with the ratio aH/f, where *H* is the height, in this case 2478 mm, and *f* the PSD lens focal length, being 7.08 mm.

**Figure 13:**
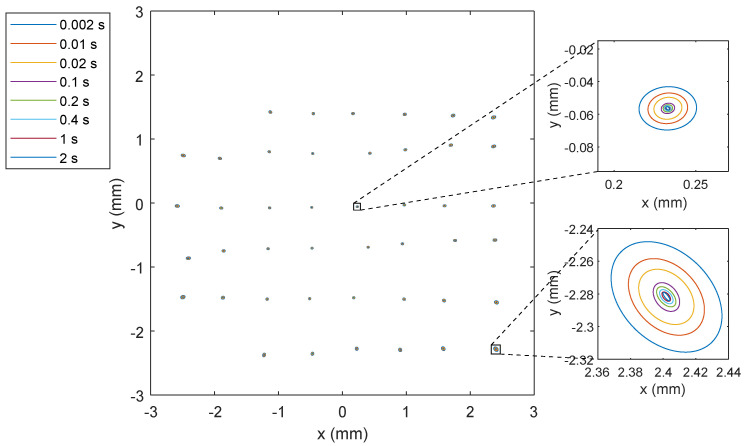
Error ellipsoids on the surface of the PSD as a function of measurement time.

The obtained precision is better than 1 mm when integrating over 1 s, whereas it degrades to 5.9 mm when the integration time is reduced to 10 ms. Figure 13 shows some examples of the size and shape of the ellipsoids on the surface of the PSD as a function of the measurement time. As expected and can be seen in the figure, the errors are larger and less circular for less central impact points on the PSD.

An additional test was carried out to analyze the degradation of precision with distance between the emitter and receiver. The emitter was fixed in one position, and the receiver was elevated across several positions while keeping the emitter in the center of its FoV. A signal was acquired at the receiver in a total of 12 distances ranging from 565 to 2765 mm. For each distance, the largest semi-majoraxis of the error ellipsoid containing 95% of the measurements was calculated using a rate of 200 measurements per second.

**Figure 14:**
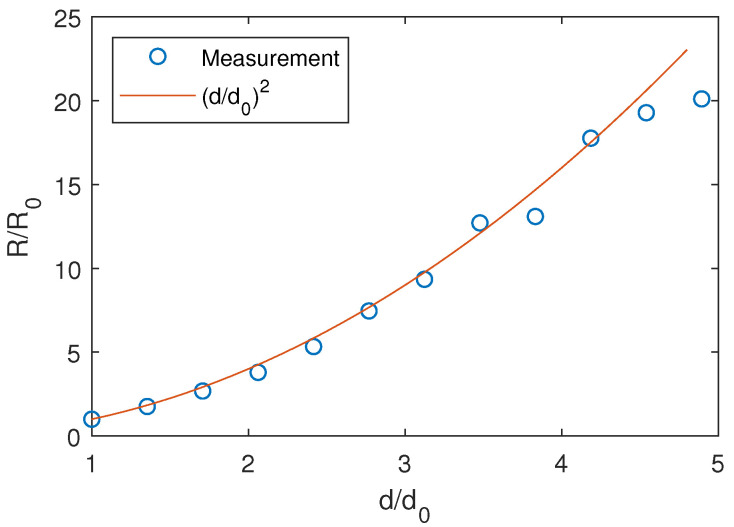
Normalized semi-major axis of the error ellipsoid as a function of distance.

Figure 14 shows the relative increase ($R/R_{0}$) of the semi-major axis as a function of distance distance, normalized to the axis value $R_{0}$ at the closest distance $d_{0}$ = 565 mm. The distance of the axis is similarly normalized to $d_{0}$. Together with the measurements, the curve representing a degradation of precision with the square of the distance is shown. This curve represents the expected reduction of the optical signal with distance due to the reduction of the solid angle covered by the PSD assuming punctual emitters—which is reasonable for the evaluated geometry—being an indicator of the theoretically expected degradation of the SNR, hence precision, with distance. A relatively high agreement with the expected values can be seen in the results. The measurement precision could be improved by increasing the emitted power and/or the effective area of the receiver with a suitable optical system.

## 6. Conclusions

An IPS based on optical signals with a PSD sensor is presented, including different positioning strategies depending on the number of visible emitters in the FoV of the receiver. An experimental accuracy and precision assessment of the proposed IPS was carried out using high-end geodetic equipment as a reference for the ground truth in an environment of 2.47 m in height covering approximately 2.2 $m^{2}$.

Accuracy is evaluated for different numbers of available emitters by using sufficiently large integration times so as to reduce random contributions to negligible levels. Positioning with one single emitter yields errors smaller than 2 cm across the complete positioning scenario, with an average error of 8.2 mm. This configuration, although of reduced system complexity, requires external measurements of the receiver rotation and the height between the emitters and the receiver movement planes, the former being non-trivial to obtain accurately, hence potentially significantly degrading the final positioning accuracy. When the signal from two emitters is available, it is possible to calculate the separation between the emitters and the plane of movement of the receiver, as well as its rotation. If the receiver moves along a plane, which is typical for many robotics applications, this allows computing its position and orientation without additional prior knowledge. The mean positioning and rotation errors measured in this configuration are 9.44 mm and 0.157°, respectively. When more than three emitters are available, the complete 3D pose (3D position and orientation) of the receiver can be calculated free of any prior knowledge or assumption on the receiver movement. The mean error obtained in this configuration is 4.87 cm, which could be improved by introducing information on the movement plane of the receiver if restricted and available.

The assessed accuracy is likely dominated by systematic errors due to the distortion of the lens system not being fully corrected, drifts in the electronics of the amplification stages that were modified due to the electrical calibration of the system, and multipath effects due to reflections of the optical signals on elements of the environment. The first two could be corrected to a large degree if needed by better calibration measurements. The latter, however, is a critical source of inaccuracy in optical IPSs. When the environment remains unchanged, partial compensation could be introduced by applying the model presented in [27] after characterization of the geometry and material composition of the environment. This, however, would require significant effort; multipath errors are therefore likely to remain the dominant source of error in most real applications.

The positioning precision is also analyzed as a function of measurement time, the distance between emitters and the receiver, and the position of the impact point within the PSD area. The results show that a precision of 5.9 mm (semi-major axis of the 2σ ellipsoid) can be achieved with integration times as small as 10 ms, improving down to below 1 mm when integrating over 1 s. As expected, the precision of the information from each emitter decreases with the square of the distance between the emitter and receiver, and the circularity of the error ellipsoid decreases for impact points further away from the PSD center.

The joint accuracy and precision assessment show that, if the application allows integration times larger than 40 ms, the overall error would be dominated by systematic deviations. In this condition and considering the evaluated scenario on a static target, the proposed IPS would yield positioning errors smaller than 3 mm in 95% of the cases. Although a fair quantitative comparison with related works is hard due to the diversity of technologies and mostly the lack of standardized test scenarios and metrics, the results of the present proposal are generally comparable to the best results of the state-of-the-art of systems using AoA or image sensors as collected in [13] and in our selected literature as revised in the Introduction. The proposed system is therefore an attractive alternative for applications, such as mobile robotics, requiring relatively high precision on fast dynamics and where there exist certain known restrictions on the possible positions of the agent to locate.

Our main proposal for future works aims at increasing performance for more demanding applications requiring 3D positioning, such as UAV localization, by investigating more optimal emitter configurations that not only use ceiling lighting, but also additional out-of-plane emitters, providing increased geometrical strength, hence potentially better accuracy.

## Figures and Tables

**Table 1:** Coordinates of the emitters’ positions.

Index of Emitter	Emitted Frequency (kHz)	x (mm)	y (mm)	z (mm)
1	6	3557	5533	2275
2	8	3758	4893	2274
3	10	3235	4395	2274
4	14	4377	4642	2271

**Table 2:** Positioning error using one emitter.

Emitter Index	Mean Error	Std Deviation Error	Maximum Error
2 (center)	8.2 mm	4.7 mm	21.8 mm
3 (corner)	10.7 mm	6.8 mm	35.8 mm

**Table 3:** Positioning error using two emitters.

	Error Using Calculated Height			Error Using Known Height		
**Indexes of the Emitters**	**Mean**	**Std**	**Max**	**Mean**	**Std**	**Max**
2–4	15.1 mm	6.3 mm	31.3 mm	8.9 mm	5.8 mm	27.1 mm
3–4	11.8 mm	6.3 mm	27.7 mm	9.2 mm	5.2 mm	25.1 mm

**Table 4:** Errors in the calculation of the rotation angle using Emitters 1 and 4.

Mean Error	Std Deviation Error	Maximum Error
0.157°	0.146°	0.440°

**Table 5:** Positioning errors using the 4 emitters.

	Mean Error	Std Deviation Error	Maximum Error
CPC	48.7 mm	35.7 mm	115 mm

**Figure A1:**
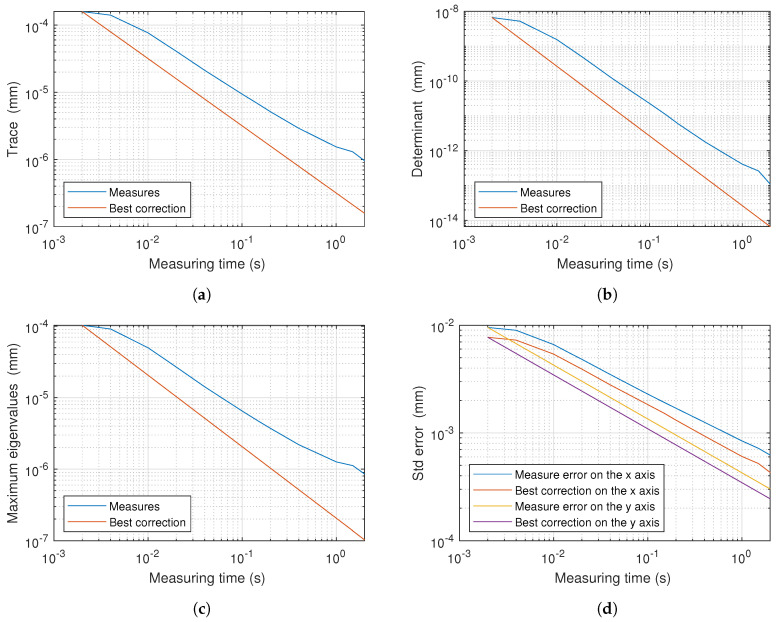
Variation of the mean value of the (**a**) trace, (**b**) determinant, and (**c**) maximum eigenvalue and (**d**) the standard deviation of the error on the x and y axes, as a function of the measurement time.

## Appendix A

In this Appendix, the trace, determinant, and maximum eigenvalues obtained from the covariance matrix of the experimental results are analyzed as a function of the measuring time:

- The trace is calculated as the sum of the eigenvalues of the covariance matrix. It gives information on the mean squared error.
- The determinant is calculated as the product of the eigenvalues. It is related to the volume of the ellipsoid.
- The maximum eigenvalue gives information on the major axis of the ellipsoid containing the point cloud.

Since measurements were obtained at several receiver positions, the values are shown by averaging them all, for each measuring time.

Figure A1a–c shows the trace, determinant, and maximum eigenvalue as a function of the measuring time. The values shown were calculated with the impact point cloud on the surface of the PSD. In theory, if the system noise were white Gaussian noise, the variance values of a signal would be reduced with the measuring time. If for example a variance value of two units is obtained with a time of 0.1 s, if 1 s is used, the variance value would be $\frac{2}{1/0.1}$. In the case of the trace and the maximum eigenvalue, this is reduced with the measurement time, whereas in the case of the determinant, this is reduced with the square of the measurement time. Figure A1d shows the variation of the mean value of the standard deviations of the error in the *x* and *y* axes as a function of the measurement time. In this case, as the standard deviation is shown, the values should be reduced by the square root of the measurement time.

Since the noise is not white Gaussian noise, the reduction in the values is less than ideal.
